# Molecular Characterization of Tobacco Streak Virus, Beet Ringspot Virus, and Beet Ringspot Virus Satellite RNA from a New Natural Host, *Phlox paniculata*

**DOI:** 10.3390/plants14111619

**Published:** 2025-05-26

**Authors:** Elena Motsar, Anna Sheveleva, Fedor Sharko, Kristina Petrova, Natalia Slobodova, Ramil Murataev, Irina Mitrofanova, Sergei Chirkov

**Affiliations:** 1 Department of Virology, Faculty of Biology, Lomonosov Moscow State University, 119234 Moscow, Russia; elena.motsar31@gmail.com (E.M.); anncsh@yandex.ru (A.S.); ramil.murataev@mail.ru (R.M.); 2 National Research Center “Kurchatov Institute”, Moscow 123182, Russia; fedosic@gmail.com (F.S.);; 3 Faculty of Biology and Biotechnology, HSE University, 101000 Moscow, Russia; nv.slobodova@gmail.com; 4 Tsitsin Main Botanical Garden of Russian Academy of Sciences, 127276 Moscow, Russia; irimitrofanova@yandex.ru

**Keywords:** ornamental plants, phlox, virome, ilarvirus, nepovirus, high-throughput sequencing, phylogenetic analysis, recombination

## Abstract

Phlox are ornamentals of great decorative value, grown throughout the world for their attractive flowers. Phlox cultivar collections at the Tsitsin Main Botanical Garden and the Botanical Garden of Lomonosov Moscow State University (both Moscow, Russia) were surveyed for virus diseases. Tobacco streak ilarvirus (TSV), beet ringspot nepovirus (BRSV), and BRSV satellite RNA (satRNA) were first detected in phlox when viromes of symptomatic *Phlox paniculata* plants were studied using high-throughput sequencing. The nearly complete genomes of three TSV and BRSV isolates and two BRSV satRNAs were assembled and characterized. TSV isolates shared 96.9–99.7% nucleotide sequence identity and were 82.2–89.1% identical to their closest relatives from broad bean, dahlia, and echinacea. BRSV isolates were distantly related to each other (83.7–89.3% identity) and were closest to those from oxalis and potato. BRSV satRNAs shared 90.6% identity and were 87.8–94.1% identical to satRNAs associated with tomato black ring virus L and S serotypes. Thus, TSV, BRSV, and BRSV satRNA were for the first time detected in a new natural host *P. paniculata* in Russia, adding to the list of known phlox viruses and expanding information on the host range, geographic distribution, and genetic diversity of these viruses.

## 1. Introduction

Phlox (genus *Phlox*, family *Polemoniaceae*) are perennial or rarely annual ornamentals of great decorative value. The genus contains about 70 species that have adapted to a wide variety of environmental conditions in the wild. First discovered in North America, phlox is now grown throughout the world as a popular landscape, garden, cut flower, and pot plant for its attractive flowers [1].

Single and mixed virus infections are common in phlox and can reduce the aesthetic appeal of infected plants. Approximately two dozen viruses of this plant, belonging to different taxonomic groups, have been recognized to date. These include tobacco ringspot, tomato ringspot, raspberry ringspot, and arabis mosaic nepoviruses [2,3,4]; strawberry latent ringspot sadwavirus [2]; alfalfa mosaic alfamovirus [5,6]; tomato spotted wilt tospovirus [7]; alternanthera mosaic potexvirus [8]; tobacco rattle tobravirus and cucumber mosaic cucumovirus [9,10]; phlox S, B, M, and ligustrum necrotic ringspot carlaviruses [4]; angelonia flower break carmovirus [11]; bidens mottle and spiranthes mosaic virus 3 potyviruses [4,12,13]; tobamovirus [4]; and phlox pilosa rhabdovirus [14]. Many phlox species were shown to be susceptible to the listed viruses.

While studying the virome of garden phlox (*P. paniculata*) plants with virus-like symptoms using high-throughput sequencing (HTS), we detected tobacco streak ilarvirus (TSV), beet ringspot nepovirus (BRSV), and BRSV satellite RNA (satRNA)-related reads in some plants.

TSV belongs to subgroup 1 of the genus *Ilarvirus* from the family *Bromoviridae*. Its segmented genome consists of three single-stranded positive-sense RNAs, each of which is packed into separate isometric particles. The 5′-end of genomic RNAs is capped, and the 3′-terminal sequence is organized into a series of stem-loops interspersed with unpaired sequences. Five open reading frames (ORFs) encode replicase, which includes methyltransferase (MET) and helicase (HEL) domains (ORF1), RNA-dependent RNA polymerase (RdRp, ORF2a), 2b protein (ORF2b), movement protein (MP, ORF3a), and coat protein (CP, ORF3b). The replicase and RdRp ensure the replication of the viral genome. The 2b protein may be involved in gene silencing and long-distance transport of the virus. The MP provides cell-to-cell transport and the CP is responsible for encapsidation of the viral genome, its systemic spread across the whole plant, and activation of the infection by binding to the 3′-terminal region of genome RNAs. TSV is transmitted between plants by seed, pollen, thrips, and vegetative propagation [15].

BRSV belongs to the genus *Nepovirus* (family *Secoviridae*), subgroup B [16,17,18]. This virus has long been considered strain S (“Scotland”) of tomato black ring virus (TBRV), which originates from burdock (*Arctium lappa*). However, BRSV is now recognized as a distinct member of the genus *Nepovirus* [19,20]. Its bipartite genome consists of two positive-sense, single-stranded RNAs. Each RNA is covalently bound to a viral genome-linked protein (VPg) at the 5′-end, has a poly (A) tail at the 3′-end and encodes a polyprotein that is processed into mature proteins by the viral protease (Pro). The RNA1-encoded polyprotein P1 is cleaved into the proteins involved in replication (HEL and RdRp), VPg, Pro and protease cofactor (Pro-Cof). The RNA2-encoded polyprotein P2 is cleaved to the CP, MP, and a 5′-proximal protein of unknown function [16,20].

BRSV is a soil-borne virus that is non-persistently transmitted by the root-feeding nematode *Longidorus elongatus* [21], and by pollen and seeds [22]. BRSV has been found in many plant species, including sugar beet, potato, turnip, wheat, oat, strawberry, raspberry, peach, and many weeds [23]. Recently, this virus was reported to infect *Euonymus alatus* and *Oxalis triangularis* in the USA [24,25] and *Begonia ricinifolia* in Hungary [26]. Complete genome sequences of BRSV from pelargonium (MZ202337, MZ202338) and raspberry (MW961152, MW961153) from Russia and Germany, respectively, have been deposited in GenBank (unpublished).

SatRNAs are often associated with nepovirus infections and can be encapsidated together with helper virus genomic RNAs. Linear type B satRNAs are of 1.1–1.5 kb in length. They are linked to a VPg at the 5′-end, have a poly(A) tail at the 3′-end, and encode a non-structural protein of 36–48 kDa that is essential for satRNA replication in cooperation with helper nepovirus replicase [16]. To date, over thirty full-length TBRV satRNA sequences have been deposited in GenBank. Some of them were detected in plants infected with TBRV S or closely related L (’Lanarkshire’) serotypes [27], both currently considered BRSV [19].

The objectives of this work were to assemble, annotate and characterize the complete genomes of the Russian TSV, BRSV, and BRSV satRNA isolates from phlox and to compare them with isolates of these viruses from other hosts.

## 2. Results and Discussion

### 2.1. HTS Results

**Figure 1:**
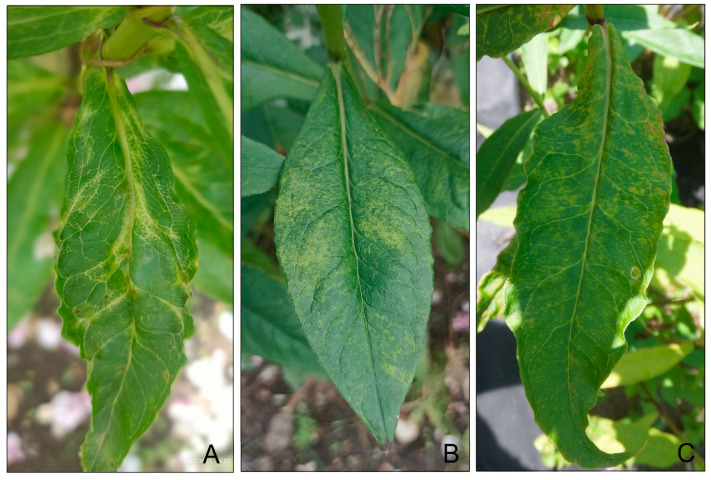
Virus-like symptoms on the leaves of the samples Px15, cultivar ‘Mirage’ (**A**) and Px31, cultivar ‘Regina’ (**B**) from the phlox cultivar collection of the Tsitsin Main Botanical Garden, and the sample PxBG2 of unknown cultivar (**C**) from the Botanical Garden of Lomonosov Moscow State University, Moscow, Russia.

During surveys of the phlox cultivar collections at the Tsitsin Main Botanical Garden of the Russian Academy of Sciences and the Botanical Garden of the Lomonosov Moscow State University, both located in Moscow, Russia, many plants with foliar virus-like symptoms were detected. Three symptomatic samples, designated Px15, Px31, and PxBG2 (Figure 1), were selected for virome analysis using HTS.

In each sample, 35–50 million pair-ended reads of 150 bp length were generated, from which 10–45 thousand contigs were assembled (Table S1). TSV- and BRSV-related contigs, covering almost the entire genome of these viruses, were identified in all three samples using NCBI BLAST v.2.15.0. Contigs related to BRSV satRNA were also assembled in the Px15 and PxBG2 samples. The depth of coverage ranged from 14× to 114× for BRSV, 13× to 208× for BRSV satRNA, and 192× to 4483× for TSV-related contigs. The raw pair-ended reads were deposited in the NCBI Sequence Read Archive (https://www.ncbi.nlm.nih.gov/sra/PRJNA1244124, accessed on 31 March 2025).

**Figure 2:**
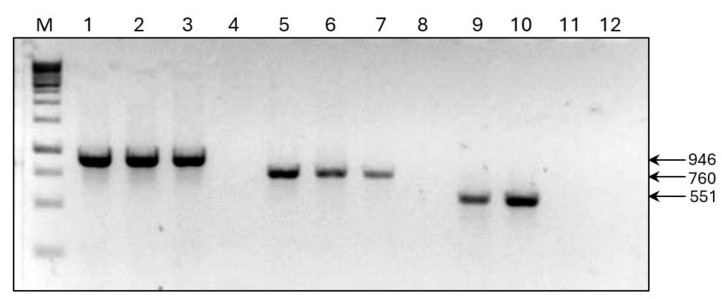
Agarose gel electrophoresis of amplicons obtained by RT-PCR assay of the samples Px15 (lanes 1, 5, 9), PxBG2 (lanes 2, 6, 10), Px31 (lanes 3, 7, 11), and negative control (uninfected plant, lanes 4, 8, 12) using virus-specific primers to tobacco streak virus (1–4), beet ringspot virus (5–8), and beet ringspot virus satellite RNA (9–12). M—GeneRuler 1 kb DNA ladder (Thermo Scientific, Waltham, MA, USA). The arrows to the right of the figure indicate PCR products of the corresponding size (bp).

The presence of these viruses in the samples was confirmed by RT-PCR using virus-specific primers designed based on nucleotide (nt) sequences of the corresponding contigs (Table S2). PCR products of the expected size of 946 (TSV), 760 (BRSV), and 551 (BRSV satRNA) bp were obtained (Figure 2). The sequences of the PCR products determined by the Sanger method were identical to the corresponding genome regions sequenced by HTS. This is the first finding of TSV, BRSV, and BRSV satRNA in phlox in Russia. In addition, reads related to alfalfa mosaic virus and tobacco rattle virus, previously reported from phlox [5,6,9], were also generated in the analyzed samples. Co-infection with different viruses is apparently responsible for the observed foliar symptoms. The differences in symptoms may be due to varietal characteristics of the infected plants, or to the different compositions of the viruses infecting them.

### 2.2. TSV Genome Characterization

Contigs related to TSV genomic RNAs were identified in each of the Px15, Px31, and PxBG2 samples.

In PxBG2, the contig of 3466 nt was most closely related (89.4% identity) to RNA1 of the Sudan DSMZ PV-0903 isolate (PP400331) from *Vicia faba*, Dutch DSMZ PV-0928 isolate (OR477283) from *Dahlia* sp., and US dp isolate (KR017708) from *D. pinnata*. ORF1 3288 nt long encoding replicase of 1055 amino acid (aa) residues was found in the contig. The MET (pfam01660) and HEL (pfam01443) domains were predicted at aa positions 68–409 and 803–1063, respectively. Another contig of 2891 nt was most closely related (87.4% identity) to RNA2 of the Chinese CNB isolate (OR183776) from *Echinacea purpurea*, Dutch DSMZ PV-0928 isolate (OR477284) from *Dahlia* sp., and US dp isolate (KR017709) from *D. pinnata*. ORF2a 2391 nt long and overlapping ORF2b 624 nt long, encoding proteins of 796 aa and 207 aa, respectively, were found in the contig. Bromoviridae RdRp motifs (cd23252) were identified at aa positions 250–621 of the ORF2a-encoded protein, including GDD motif at positions 554–556. One more contig of 2183 nt was most closely related (89.1% identity) to RNA3 of the Dutch DSMZ PV-0909 isolate (OR082771) from impatiens, Dutch DSMZ PV-0928 isolate (OR477285) from dahlia, and US dp isolate (KR017710) from *D. pinnata*. ORF3a and ORF3b of 870 nt and 714 nt, encoding the Bromovirus MP (cI03270) and Ilarvirus CP (pfam01787) of 289 aa and 237 aa, respectively, were found. The ATGC and arginine-rich motifs involved in genome activation [15] were identified at the 3′-end of genomic RNAs and at the N-terminus of the CP, respectively. ORF3a and ORF3b were separated by the intergenic region of 123 nt. Sequence analysis showed that these three contigs appeared to correspond to TSV genomic RNAs.

Characterization of TSV isolates from other samples revealed that all three (designated TSV-Px15, TSV-Px31, and TSV-PxBG2) were very similar (Table 1). MegAlign multiple alignments showed that their RNA1, RNA2, and RNA3 were 99.7%, 96.9%, and 99.4% identical to each other, respectively. At the same time, they shared 88.6–89.1% (RNA1), 82.8–87.0% (RNA2), and 87.9–88.8% (RNA3) identity at the nt level with their closest relatives from other hosts. At the aa level, five ORFs of the phlox isolates were 96.6–100% identical to each other. With their closest relatives, they shared 93.0–93.4% (ORF1), 87.9–88.7% (ORF2a), 86.6–88.8% (ORF2b), 79.9–87.2% (ORF3a), and 90.7–92.8% (ORF3b) aa identity. The 3′-untranslated regions (UTR) of RNA1, RNA2, and RNA3 were identical among the three virus isolates, and the 5′-UTRs were also similar.

Compared to the closest relatives from other hosts, replicase of the phlox TSV isolates was two aa longer due to triplet insertions at ORF1 positions 746–748 and 1515–1517 (coding for glutamate and lysine, respectively). Two gaps of two and six nt length were found in the 3′-UTR of RNA1. RdRp was one aa (aspartate) longer due to triplet insertion to the RNA2 overlapping genome region. The 2b protein was two aa longer due to the above triplet insertion and because its start AUG codon is shifted one triplet upstream, so that there are two methionine residues in a row at the N-terminus of this protein. Such a methionine tandem is common in the ilarvirus 2b protein, but for TSV it was only found in the phlox-adapted isolates and US isolate OK (KP256251) from soybean. MP was one aa (serine) shorter due to the corresponding triplet deletion in the C-terminal part of ORF3a. Deletions in the C-terminus of the MP have been suggested to influence host specificity of ilarviruses [28]. The 2b protein and MP are considered to be involved in the systemic transport of ilarviruses throughout the plant [15]. The MP and 2b protein features common to all three TSV isolates, together with the relatively low aa identity of these proteins with their counterparts from other hosts, may possibly be related to the TSV adaptation to phlox. The genome RNA sequences of TSV-Px15, TSV-Px31, and TSV-PxBG2 isolates were deposited in GenBank under accession numbers PV524995–PV525003.

**Figure 3:**
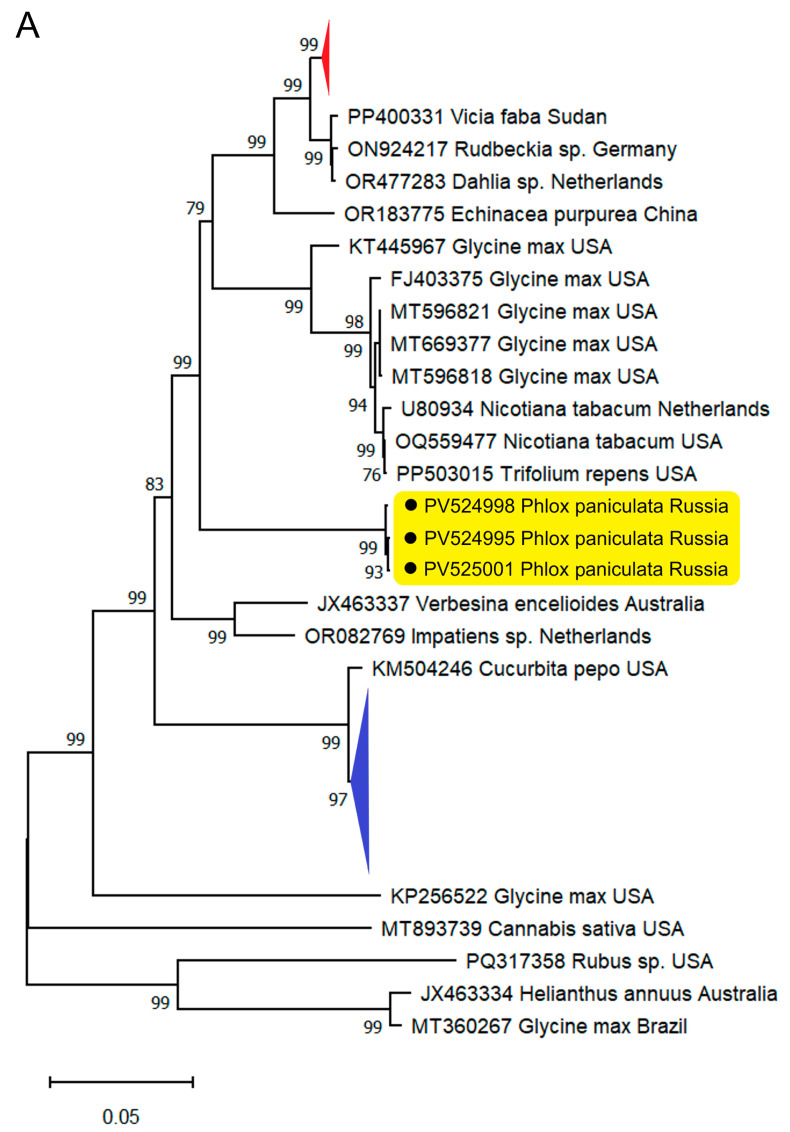

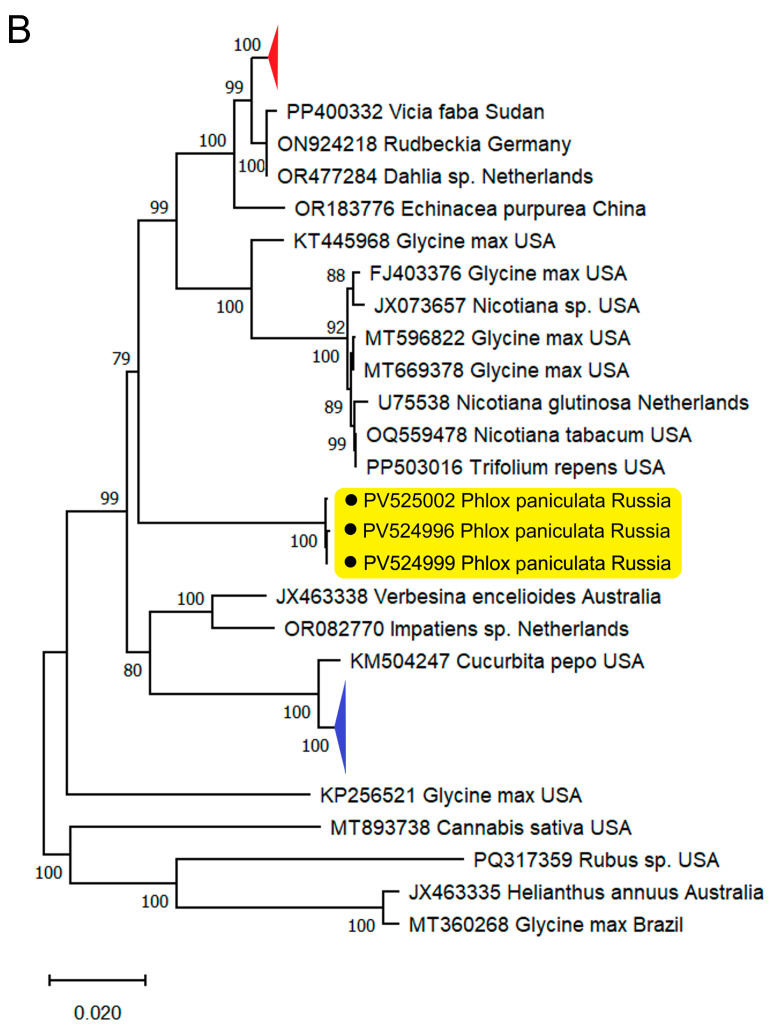

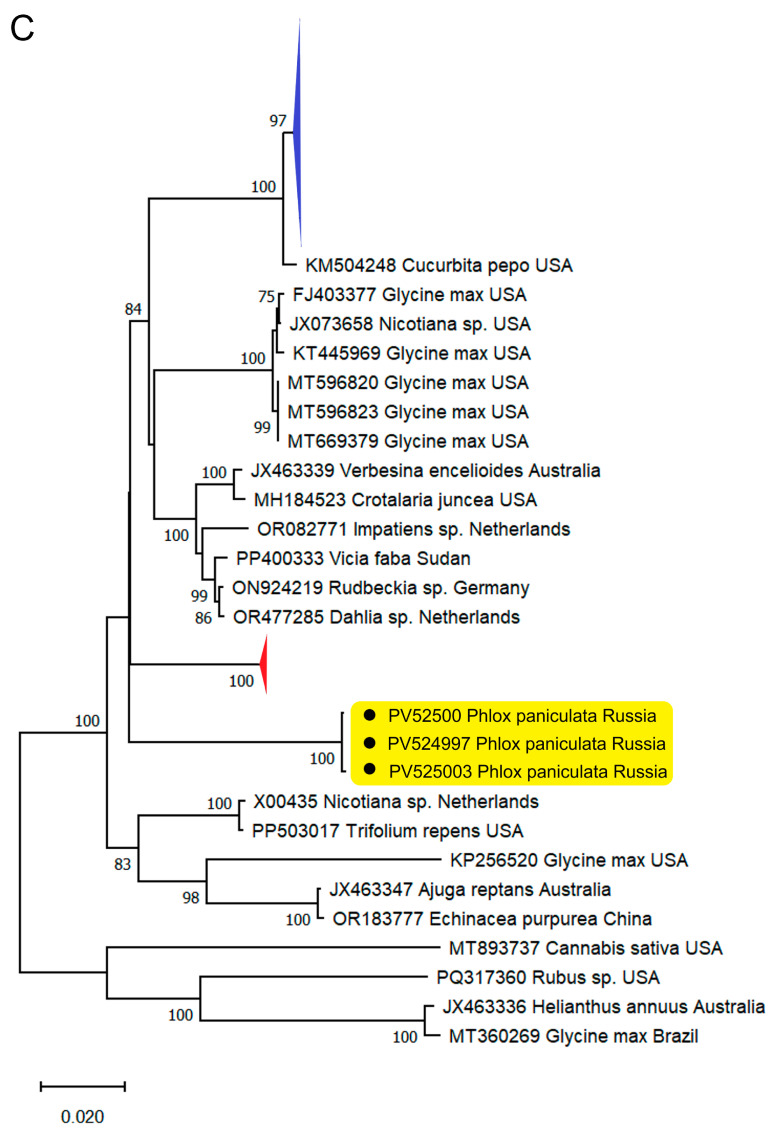
Maximum likelihood phylogenetic trees based on full-length sequences of tobacco streak virus (TSV) genome RNA1 (**A**), RNA2 (**B**), and RNA3 (**C**) using the GTR best-fit substitution model. The accession number, Latin name of the host plant, and geographical origin of the virus isolate are given at the end of branches. Russian phlox isolates are in yellow and marked with a black circle (●). Blue and red triangles denote condensed clusters represented with Indian isolates from different hosts and a group of US isolates from soybean, respectively. Bootstrap values from 1000 replicates (>75%) are shown next to the corresponding node. The scale bar indicates the number of nucleotide substitutions per site.

Phylogenetic analysis of TSV genomic RNAs showed that the phlox isolates formed a distinct clade in each of the trees, as supported by the high bootstrap values (Figure 3). Both tree topology and position of this clade within the tree were similar for each genome segment, suggesting that there was no intraspecies recombination or reassortment in the phlox virus isolates. No recombination was detected in the phlox TSV isolates using the Recombinant Detection Program (RDP) v.4.101 [29].

**Figure 4:**
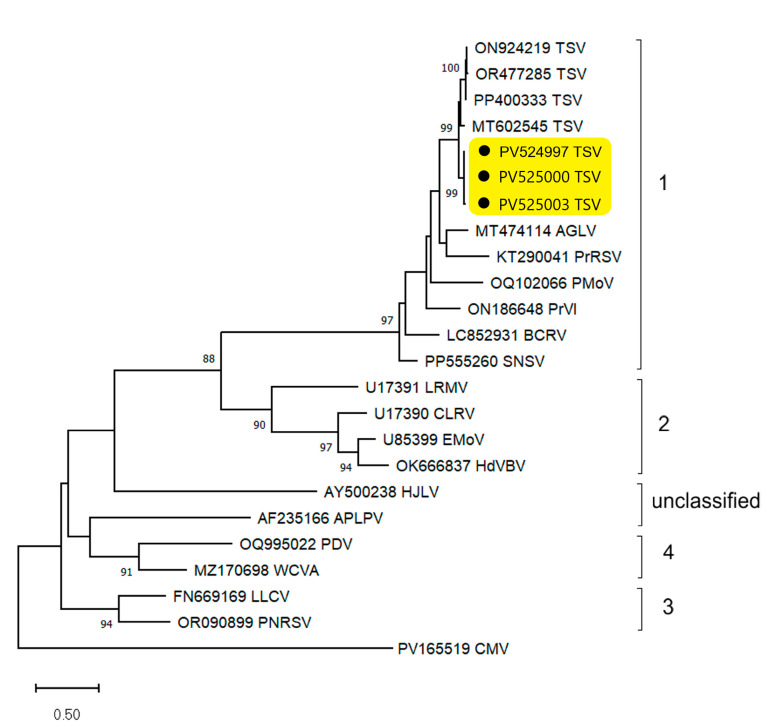
Phylogenetic analysis of tobacco streak virus (TSV) isolates from phlox and selected members of the genus *Ilarvirus* based on the alignment of the complete amino acid sequences of the coat protein. The tree was reconstructed in MEGA X using the maximum likelihood method with the WAG best-fit substitution model. Bootstrap values (>85%) from 1000 replicates are shown next to the corresponding nodes. The accession numbers in GenBank and virus acronyms are shown at the end of branches. Brackets combine isolates of the same subgroup. The scale bar indicates the number of substitutions per nucleotide. The phlox TSV isolates are highlighted with yellow and a black circle (*•*). Cucumber mosaic virus (CMV) was used as an outgroup. Viruses used for tree reconstruction are (subgroup 1) AGLV—ageratum latent virus, BCRV—blackberry chlorotic ringspot virus, PMoV—parietaria mottle virus, PrRSV—privet ringspot virus, PrVI—prunus virus I, and SNSV—strawberry necrotic shock virus; (subgroup 2) CLRV—citrus leaf rugose virus, EMoV—elm mottle virus, HdVBV—hydrangea vein banding virus, LRMV—lilac ring mottle virus; (subgroup 3) LLCV—lilac leaf chlorosis virus, PNRSV—prunus necrotic ringspot virus; (subgroup 4) PDV—prune dwarf virus, WCVA—water chestnut virus A; (unclassified) APLPV—American plum line pattern virus, and HJLV—humulus japonicus latent virus.

The criteria used to distinguish species within the genus *Ilarvirus* are serology, host range, and sequence similarity, although specific levels of sequence identity have not been defined [30]. As with TSV, the MP and CP of isolates from different hosts and locations show more than 84% and 95% identity at the aa level, respectively [15]. Additionally, these proteins from the phlox TSV isolates were only 79.9–87.2% (MP) and 90.7–92.8% (CP) identical to their counterparts from even the closest isolates of this virus. On the other hand, phylogenetic analysis of the ilarvirus CPs aa sequences showed that the phlox TSV isolates grouped together with isolates of this virus from other hosts, which formed a sister clade, but separately from the other viruses in subgroup 1 (Figure 4). Thus, the phlox TSV isolates represent apparently divergent forms of this virus. This is the first detection of TSV and ilarvirus in general in phlox.

### 2.3. BRSV Genome Characterization

Contigs related to BRSV genomic RNA1 and RNA2 were assembled in each of the Px15, Px31, and PxBG2 samples. In PxBG2, a single ORF of 6804 nt encoded a polyprotein P1 of 2267 aa was identified in contig of 7327 nt long. This polyprotein was predicted to be cleaved at specific dipeptide sites to produce five mature proteins (Table 2). The prediction was based on analysis of the polyprotein processing sites in other BRSV isolates and subgroup B nepoviruses [16]. The conserved HEL (pfam00910) and RdRp (cI40470) domains were found at P1 polyprotein aa positions 778–879 and 1637–1965, respectively. Single ORF of 4071 nt encoded a polyprotein P2 of 1356 aa was found in another contig of 4776 nt in the PxBG2 sample. The P2 polyprotein was predicted to be cleaved to produce the CP and MP (Table 2). The cleavage site upstream from MP is still uncertain. The N-terminal (pfam03689), central (pfam03391), and C-terminal (pfam03688) conserved nepovirus CP domains were found in the P2 polyprotein at aa positions 842–932, 1007–1170, and 1176–1337, respectively.

BRSV isolates from Px15, Px31, and PxBG2 samples were similar in RNA and ORF length, genome organization (Table 3), and cleavage site composition (Table 2). Only the BRSV-Px15 P2 polyprotein is one aa shorter due to a glutamate-encoding GAG triplet deletion at positions 2477–2479. Phlox BRSV isolates were shown to be distantly related to each other. Their RNA1 shared 85.0–89.3% nt and 91.9–95.1% aa identity, while RNA2 shared 83.7–86.7% nt and 90.9–93.1% aa identity, respectively. In addition, RNA2 of PxBG2 and Px31 are the longest among the RNA2s of this virus, except the isolate DSMZ PV-0521 (MZ202338) from pelargonium. In contrast, Px15 RNA2 is the shortest due to several long gaps in the 5′ UTR.

Also, BLAST showed different nearest relatives for both RNAs from each of the three phlox isolates. RNA1 of the BRSV-PxBG2 and BRSV-Px15 isolates were most closely related (89.6% identity) to the US isolate Ox (MH939189) from oxalis, while BRSV-Px31 RNA1 was closest to the UK isolate IF (OM640126) from potato (86.1% identity). RNA2 of the BRSV-PxBG2 and BRSV-Px31 isolates were most closely related (94.6% and 87.5% identity, respectively) to the Dutch isolate BRox2 (MG727392) from oxalis (query cover 99% and 85%, respectively). RNA2 of the BRSV-Px15 was closest (91.9% identity) to the Russian isolate DSMZ PV-0521 (MZ202338) from pelargonium. However, the P2 polyprotein sequences of BRSV isolates Px15, Px31, and PxBG2 were all most closely related to that of the BRox2 isolate, with 91.0%, 93.3%, and 96.4% aa identity, respectively. It cannot be ruled out that the phlox BRSV isolates are closest to the BRox2 isolate, but only the ORF sequence in BRox2 RNA2 is currently available for comparison. The genome RNA sequences of the BRSV-Px15, BRSV-Px31, and BRSV-PxBG2 isolates were deposited in GenBank under accession numbers PV565076–PV565081.

**Figure 5:**
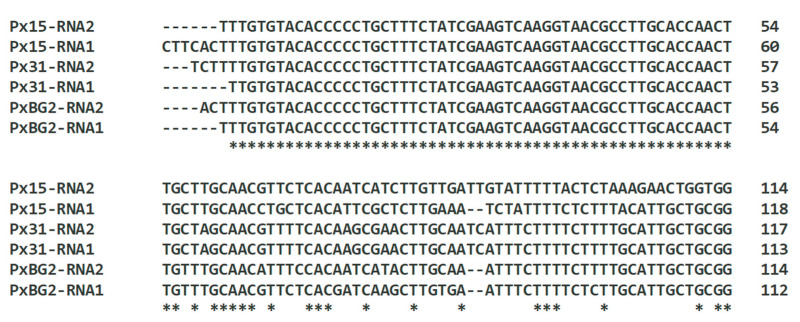
Alignment of the 5′ untranslated regions of RNA1 and RNA2 of beet ringspot virus isolates from phlox using Clustal Omega (v.1.2.4) [31]. The alignment shows the 5′-terminal regions of approximately 110 nt. *—identical nucleotide in all the virus isolates.

RNA1 and RNA2 of nepoviruses can share extended regions of high sequence identity in the UTRs [17]. This is the case for the phlox BRSV isolates, despite the relatively low genome RNA identities. The first 60–70 nt at the 5′ ends of their genome RNAs were almost identical (Figure 5). A high degree of identity was demonstrated not only for two RNAs of the same isolate, but also between all three isolates of the virus. The 3′ UTRs of RNA1 and RNA2 were 97.4–99.7% identical to one another for each of the phlox isolates.

**Figure 6:**
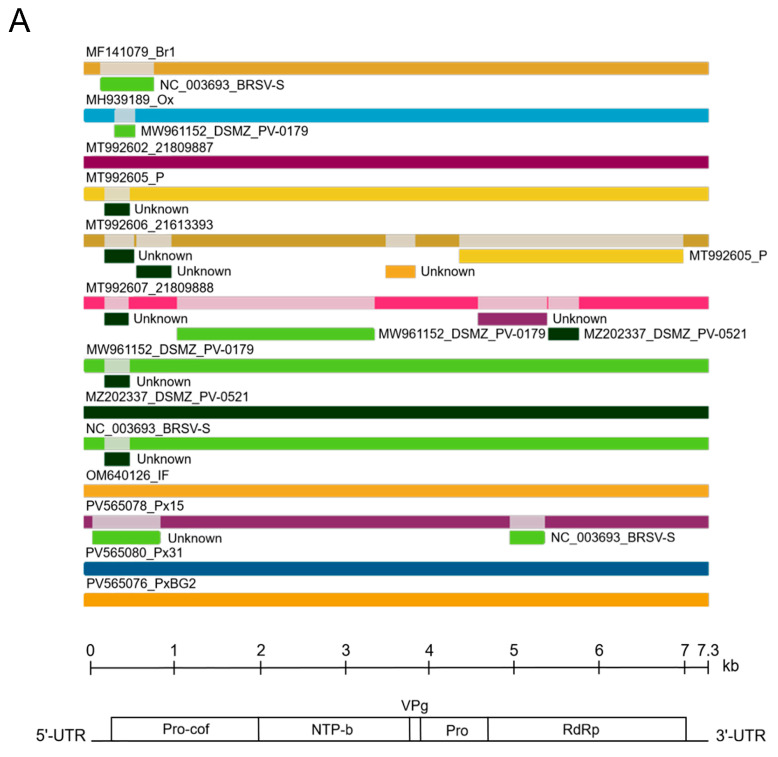

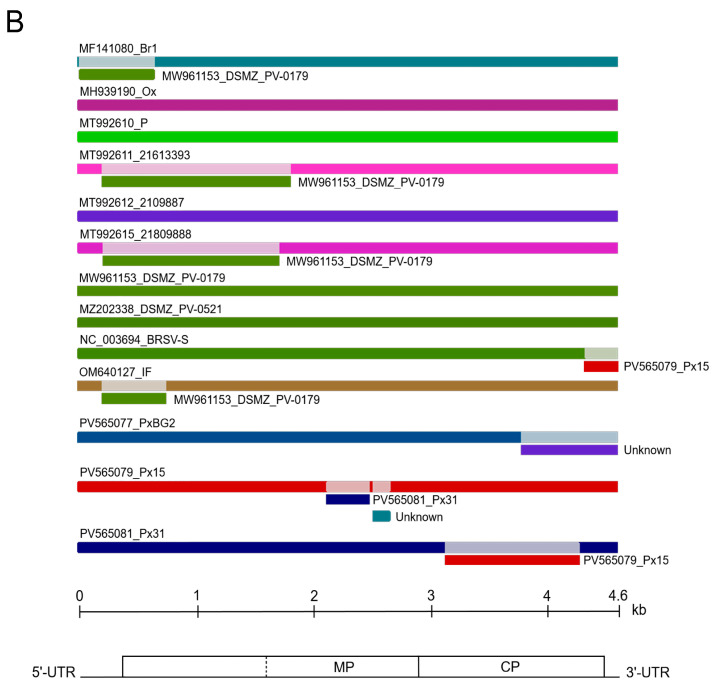
Analysis of recombination events in the alignment of full-length beet ringspot virus (BRSV) genomic RNA1 (**A**) and RNA2 (**B**) by the Recombinant Detection Program (RDP) v.4.101 [29]. Summarized results of the recombination events detection are presented. The names of isolates and their GenBank accession numbers are indicated above the long bars. The names of inferred minor parents are indicated to the right of short bars beneath the corresponding isolate bars. The map of the BRSV genome and the ruler are presented in scale below the graph. The 5′ proximal MP boundary in RNA2 is not identified and is shown on the scheme with a vertical dotted line.

Recombination occurs often in BRSV genomes [19,20]. We searched for recombination using the alignment, which included complete BRSV genome sequences available in GenBank and newly assembled in this work. Consistent with previous findings, a large number of recombination events (REs) were detected in BRSV isolates from different hosts. All three phlox isolates were also shown to be recombinants (Figure 6, Table 4). Both BRSV-Px15 RNAs were multiple recombinants. REs were revealed in the genes encoding Pro-Cof, RdRp, MP, CP, and also in the UTRs. Thus, REs were found in different genome regions, indicating no recombination hotspots. When known, inferred minor parents (parental sequences representing a minor part of the recombinant genome) were closely related to the corresponding genomic region of the recombinant, increasing the reliability of RE detection. In contrast, the inferred major parents (parental sequences representing a major part of the recombinant genome) were only distantly related to the recombinant genome sequences, indicating that the list of known BRSV isolates appears to be considerably incomplete to suggest a more appropriate major parent. Apparently, closer isolates have yet to be found or their full-length genomes are not currently available. It is also interesting to note that RNA2 of BRSV strain S (NC_003694) is a recombinant in which the major parent is the UK BRSV strain P isolate from potato (MT992610) and the minor parent is the Russian isolate Px15 from phlox (PV565079). This RE was supported with a high degree of confidence (3.320 × 10^−25^). The major and minor parents had 99.6% and 96.8% identity, respectively, with the recombinant sequence.

### 2.4. Characterization of BRSV satRNA

Contigs of 1374 nt and 1365 nt in length were assembled from the reads generated in the Px15 and PxBG2 samples, respectively. They shared 91.0% nt identity and were shown to be most closely related (87.8–94.1% identity) to satRNAs associated with TBRV serotypes L (X05687) and S (X00978) and BRSV from hosta (*Hosta* sp.) (KX033801). Since TBRV-S and TBRV-L are now considered to be BRSV [19], we named satellite RNAs found in the BRSV-infected plants as BRSV satRNAs.

**Figure 7:**
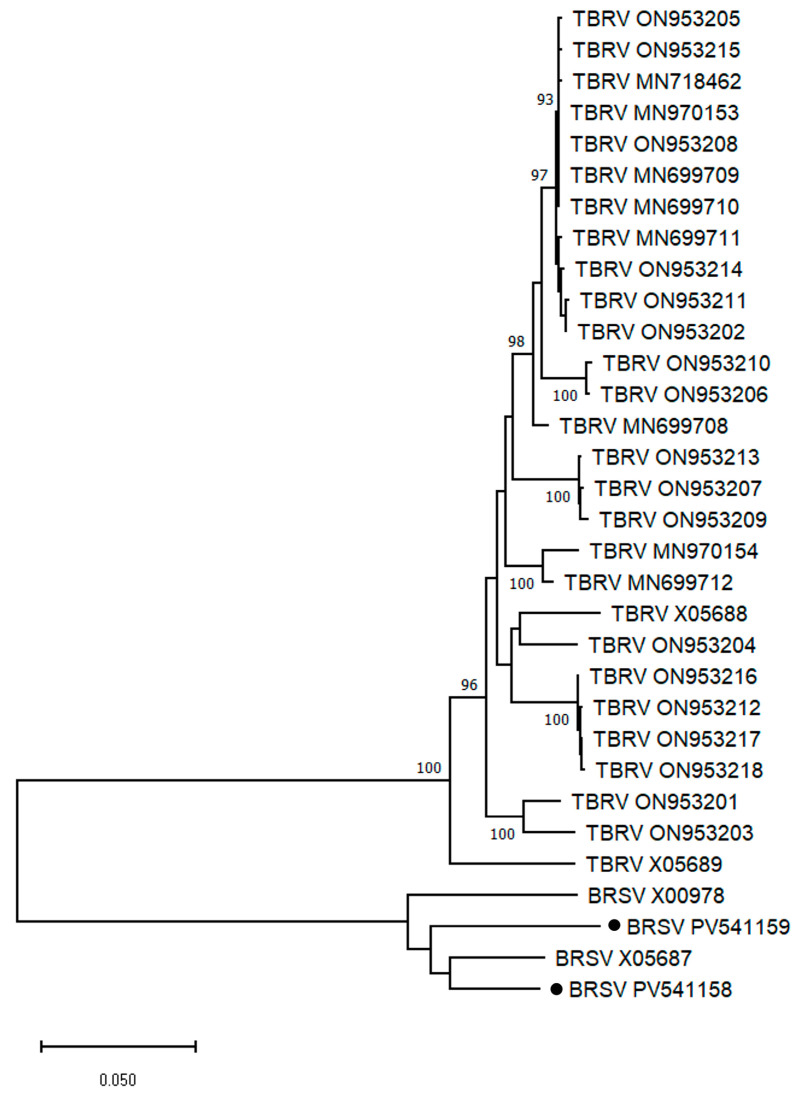
Maximum likelihood phylogenetic tree based on the complete sequences of tomato black ring virus (TBRV) and beet ringspot virus (BRSV) satellite RNAs using the GTR best-fit substitution model. The name of helper viruses and GenBank accession numbers of satRNAs are given at the end of branches. Phlox isolates are marked with a black circle (●). Bootstrap values from 1000 replicates (>80%) are shown next to the corresponding nodes. The scale bar indicates the number of nucleotide substitutions per site.

Single ORFs of 1275 nt and 1272 nt encoding proteins of 424 aa (48,022 Da) and 423 aa (47,816 Da) were predicted in the Px15 and PxBG2 contigs, respectively. Apparently, these putative proteins correspond to a non-structural 48 kDa protein encoded by other TBRV or BRSV satRNAs [16]. The PxBG2 ORF is three nt shorter due to triplet deletion at positions 964–966. ORFs were flanked by the 5′-UTRs of 12 nt and the 3′-UTRs of 87 (Px15) or 81 (PxBG2) nt. Based on molecular analysis, the Px15 and PxBG2 contigs appear to represent nearly complete satRNA sequences that were deposited to GenBank under accession numbers PV541158 and PV541159, respectively. Phylogenetic analysis of the complete TBRV and BRSV satRNAs clearly divided them into two clusters (Figure 7). The larger one is mainly represented by Polish TBRV satRNAs from different hosts. Phlox BRSV satRNAs clustered with TBRV serotypes S and L-associated satRNAs and were only 68.3–70.6% identical to TBRV satRNAs from the larger cluster.

This was the first demonstration of nepovirus satRNA in phlox. No reads associated with viral satRNAs or PCR products of the expected size were obtained in the Px31 sample, which is consistent with previous findings that satRNA does not always occur in nepovirus infection [19,20].

## 3. Materials and Methods

### 3.1. Sampling and Sample Processing

The phlox cultivar collections at the Tsitsin Main Botanical Garden of the Russian Academy of Sciences (N 55.832718; E 37.598346) and the Botanical Garden of the Lomonosov Moscow State University (N 55.844481, E 37.589794), both located in Moscow, Russia, were surveyed for virus diseases in the summer of 2023, just before phlox bloom. Leaves with suspected virus-like symptoms were collected and delivered to the Department of Virology of Lomonosov Moscow State University for further processing. Total RNA was isolated from fresh leaves using an RNeasy Plant Mini Kit (Qiagen, Hilden, Germany) according to the manufacturer’s instructions and stored at −70 °C until use.

### 3.2. High-Throughput Sequencing (HTS)

cDNA libraries were synthesized on the total RNA template using a TruSeq Stranded Total RNA Library Prep Plant kit (Illumina, San Diego, CA, USA) and sequenced on an Illumina NovaSeq 6000 platform at the National Research Center “Kurchatov Institute”. The resulting 150 nt paired-end reads were quality-checked with FastQC v0.12, then trimmed and filtered using fastp v.0.20.1 using default settings. Contigs were assembled de novo using metaSPAdes v.3.15 assembler [32]. Virus-associated contigs were identified using BLASTn v2.15.0 (https://blast.ncbi.nlm.nih.gov/Blast.cgi, accessed on 12 September 2024) against the GenBank Virus database (September 2024) with an E-value cutoff of 1 × 10^−5^ and a non-human host filter applied.

### 3.3. Reverse Transcription–Polymerase Chain Reaction (RT-PCR)

Total RNA was used as a template for RT-PCR detection of TSV, BRSV, and BRSV satRNA. The first-strand cDNA was synthesized with random hexamer primers and Moloney murine leukemia virus (MMLV) reverse transcriptase (Evrogen, Moscow, Russia). PCR amplification was performed using proofreading Encyclo DNA polymerase (Evrogen) and virus-specific primers, designed based on the nearly full-length genome sequences of TSV, BRSV, and BRSV satRNA obtained in this study (Table S2). Total RNA from uninfected phlox plants was included as a negative control. PCR products were resolved by electrophoresis in 1.5% agarose gel, stained with ethidium bromide and visualized using a MultiDoc-It imaging system (Analytik Jena US LLC, Upland, CA, USA). Amplicons of the expected sizes were gel-purified using a BC022 Cleanup Standard Kit (Evrogen) and sequenced bidirectionally at Evrogen’s sequencing facilities.

### 3.4. Sequence Analyses

For analysis of the newly assembled TSV, BRSV, and BRSV satRNA sequences, full-length genomes of other isolates of these viruses were retrieved from GenBank. Multiple sequence alignment was performed using the ClustalW algorithm implemented in the MegAlign DNASTAR Lasergene software package (version 15) [33]. The resulting alignments were used to calculate nucleotide and amino acid sequence identities between viral isolates and for phylogenetic analyses conducted in MEGA X [34]. Phylogenetic trees were reconstructed using the maximum likelihood method, with the GTR model for nucleotide-based trees and the WAG model for amino acid-based trees, selected as the best-fit substitution models. ORFs in the viral genomes were identified using the ORF Finder program (https://ncbi.nlm.nih.gov/orffinder, accessed on 29 October 2024). Conserved protein domains were identified using the Conserved Domain Database (CDD, https://ncbi.nlm.nih.gov/Structure/cdd/wrpsb.cgi, accessed on 29 October 2024). Potential recombination events in TSV and BRSV genomes were analyzed using RDP v.4.101 [29] with the default settings except that “sequences are linear” and “list events detected by >6 methods” options were chosen.

## 4. Conclusions

In identifying viruses in plant transcriptomes using HTS, TSV, BRSV, and BRSV satRNA were for the first time detected in a new natural host *P. paniculata* in Russia. To the best of our knowledge, none of these viruses have ever been previously detected in phlox. TSV is the first ilarvirus detected in this crop. The nearly complete genomes of three TSV and BRSV isolates and two BRSV satRNAs were assembled and characterized. These findings add to the list of known phlox viruses and expand information on the host range, geographic distribution, and genetic diversity of these viruses.

## Figures and Tables

**Table 1:** Length of genomic RNAs and open reading frames (ORFs) in phlox isolates of tobacco streak virus (TSV).

Genome Segment ^a^	Virus Isolates			Encoded Protein ^b^
	TSV-Px15	TSV-Px31	TSV-PxBG2	
RNA1 (nt/GenBank accession number)	3474/PV524995	3467/PV524998	3466/PV525001	
ORF1 (nt/aa)	3288/1095	3288/1095	3288/1095	MET, HEL
RNA2 (nt/GenBank accession number)	2890/PV524996	2898/PV524999	2891/PV525002	
ORF2a (nt/aa)	2391/796	2391/796	2391/796	RdRp
ORF2b (nt/aa)	624/207	624/207	624/207	2b protein
RNA3 (nt/GenBank accession number)	2196/PV524997	2191/PV525000	2183/PV525003	
ORF3a (nt/aa)	870/289	870/289	870/289	MP
ORF3b (nt/aa)	714/237	714/237	714/237	CP

**^a^** nt—nucleotides, aa—amino acids; ^b^ MET—methyltransferase; HEL—helicase; RdRp—RNA-dependent RNA polymerase; MP—movement protein; CP—coat protein.

**Table 2:** Position and length of the genes in beet ringspot virus isolate PxBG2 genome and dipeptide sequences of the polyprotein cleavage sites.

Genome Segment	Gene ^a^	Gene Positions, nt	Gene/Protein Length, nt/aa	Putative Cleavage Site
RNA1	Pro-cof	223..2043	1821/607	^607^R/S^608^
	NTP-b	2044..3840	1797/599	^1206^K/A^1207^
	VPg	3841..3921	81/27	^1233^K/S^1234^
	Pro	3922..4551	630/210	^1443^K/S^1444^
	RdRp	4552..7026	2475/824	
RNA2	MP	405..2915	2511/837	^837^K/A^838^
	CP	2916..4475	1560/519	

^a^ Pro-cof—proteinase cofactor; NTP-b—NTP-binding protein; VPg—viral genome-linked protein; Pro—cysteine proteinase; RdRp—RNA-dependent RNA polymerase; MP—movement protein; CP—coat protein.

**Table 3:** Length of genomic RNAs and open reading frames (ORFs) in phlox isolates of beet ringspot virus (BRSV).

Genome Segment ^a^	Virus Isolates			Encoded Protein
	BRSV-Px15	BRSV-Px31	BRSV-PxBG2	
RNA1 (nt/GenBank accession number)	7335/PV565078	7330/PV565080	7327/PV565076	
ORF (nt/aa)	6804/2267	6804/2267	6804/2267	Polyprotein P1
RNA2 (nt/GenBank accession number)	4601/PV565079	4745/PV565081	4776/PV565077	
ORF (nt/aa)	4068/1355	4071/1356	4071/1356	Polyprotein P2

**^a^** nt—nucleotides, aa—amino acids.

**Table 4:** Intraspecies recombination events inferred in RNA1 and RNA2 of phlox beet ringspot virus (BRSV) isolates by Recombination Detection Program v.4.101 (RDP4).

Genome Segment	Recombinant	Beginning Breakpoint	Ending Breakpoint	Major Parent ^a^	Minor Parent ^a^	*p*-Value ^b^
RNA1	BRSV-Px15	78	865	Br1 (89.4%)	Unknown	2.328 × 10^−25^
		4988	5411	Ox (90.8%)	BRSV-S (98.8%)	2.250 × 10^−10^
RNA2	BRSV-Px31	3212	4432	Unknown	BRSV-Px15 (97%)	1.820 × 10^−42^
	BRSV-Px15	2420	2574	BRSV-Px31 (88.6%)	Unknown	1.439 × 10^−22^
		2005	2400	BRSV-PxBG2 (89.6%)	BRSV-Px31 (98.2%)	5.260 × 10^−13^
	BRSV-PxBG2	3928	3′ end	DSMZ PV-0179 (91.1%)	Unknown	3.695 × 10^−09^

^a^ In parenthesis: identity shared by the corresponding genomic region in the recombinant isolate and the inferred parent. ^b^ Multiple comparison corrected *p*-values obtained from seven recombination detection methods (RDP, GENECONV, Bootscan, Maxchi, Chimaera, SiScan, 3Seq) as implemented in RDP4.

## Data Availability

Sequencing data have been deposited in NCBI Sequence Read Archive and GenBank, and their accession numbers are provided within the article.

## Supplementary Materials

The following supporting information can be downloaded at https://www.mdpi.com/article/10.3390/plants14111619/s1. Table S1: Results of high-throughput sequencing of phlox samples with symptoms of virus disease. Table S2: Virus-specific primers used for tobacco streak virus (TSV), beet ringspot virus (BRSV), and beet ringspot virus satellite RNA (BRSV satRNA) detected by RT-PCR.
