# Supplementary material for: Molecular Characterization of Tobacco Streak Virus, Beet Ringspot Virus, and Beet Ringspot Virus Satellite RNA from a New Natural Host, Phlox paniculata
Source: Plants (Basel). 2025 May 26;14(11):1619. doi: 10.3390/plants14111619 (PMC12157320; doi:10.3390/plants14111619)
Supplement: Supplementary file 1 [file plants-14-01619-s001.zip › Table S1.pdf]

Table S1. Results of high-throughput sequencing of phlox samples with symptoms of virus disease

Sample	Number of raw reads	Read length	Number of assembled contigs[a]	
			In total	Virus-related
Px15	40,262,202	150	39,011	668
Px31	35,663,968	150	45,360	645
PxBG2	51,817,462	150	10,919	218

[a]Over 300 bp
