# Supplementary material for: Molecular Characterization of Tobacco Streak Virus, Beet Ringspot Virus, and Beet Ringspot Virus Satellite RNA from a New Natural Host, Phlox paniculata
Source: Plants (Basel). 2025 May 26;14(11):1619. doi: 10.3390/plants14111619 (PMC12157320; doi:10.3390/plants14111619)
Supplement: Supplementary file 1 [file plants-14-01619-s001.zip › Table S2.pdf]

**Table S2.** Virus-specific primers for tobacco streak virus (TSV), beet ringspot virus (BRSV), and beet ringspot virus satellite RNA (BRSV satRNA) detection by RT-PCR

| Virus | Primer | Sequence, 5'..3' | Target RNA/Gene[a] | Cycling conditions (denaturation/annealing/ elongation), 35 cycles | PCR product, bp |
|---|---|---|---|---|---|
| TSV | PxTSV-CP-F | AGTCGCTTCTCGGACTTACCT | RNA3/CP | 94°C 30 s/50°C 30 s/ 72°C 1 min | 946 |
| | PxTSV-CP-R | GGAAATCGTCCGATTCGGAAT | | | |
| BRSV | PxBRSV-F | TGAACTTAAGAGCCAAATTGAATA | RNA1/RdRp | 94°C 30 s/50°C 30 s/ 72°C 50 s | 760 |
| | PxBRSV-R | GACAAGGCAAGACATGTCTATT | | | |
| BRSV satRNA | stBRSV-F | AGGGTATGTACGCCAGTTCCT | satRNA/p48 | 94°C 30 s/50°C 30 s/ 72°C 45 s | 551 |
| | stBRSV-R | GCTCTGGAGAAAAGGTATACAAT | | | |

[a]CP - coat protein; RdRp - RNA-dependent RNA polymerase
